# Novel Heptaplex PCR-Based Diagnostics for Enteric Fever Caused by Typhoidal *Salmonella* Serovars and Its Applicability in Clinical Blood Culture

**DOI:** 10.4014/jmb.2307.07031

**Published:** 2023-08-21

**Authors:** Hyun-Joong Kim, Younsik Jung, Mi-Ju Kim, Hae-Yeong Kim

**Affiliations:** 1 Department of Food Engineering, Mokpo National University, Muan 58554, Republic of Korea; 2 Institute of Life Sciences and Resources and the Department of Food Science and Biotechnology, Kyung Hee University, Yongin 17104 Republic of Korea

**Keywords:** Enteric fever, typhoidal *Salmonella* serovar, PCR, diagnostics

## Abstract

Enteric fever is caused by typhoidal *Salmonella* serovars (Typhi, Paratyphi A, Paratyphi B, and Paratyphi C). Owing to the importance of *Salmonella* serovars in clinics and public hygiene, reliable diagnostics for typhoidal serovars are crucial. This study aimed to develop a novel diagnostic tool for typhoidal *Salmonella* serovars and evaluate the use of human blood for clinically diagnosing enteric fever. Five genes were selected to produce specific PCR results against typhoidal *Salmonella* serovars based on the genes of *Salmonella* Typhi. Heptaplex PCR, including genetic markers of generic *Salmonella*, *Salmonella enterica* subsp. *enterica*, and typhoidal *Salmonella* serovars, was developed. Typhoidal *Salmonella* heptaplex PCR using genomic DNAs from 200 *Salmonella* strains (112 serovars) provided specifically amplified PCR products for each typhoidal *Salmonella* serovar. These results suggest that heptaplex PCR can sufficiently discriminate between typhoidal and nontyphoidal *Salmonella* serovars. Heptaplex PCR was applied to *Salmonella*-spiked blood cultures directly and provided diagnostic results after 12- or 13.5-h blood culture. Additionally, it demonstrated diagnostic performance with colonies recovered from a 6-h blood culture. This study provides a reliable DNA-based tool for diagnosing typhoidal *Salmonella* serovars that may be useful in clinical microbiology and epidemiology.

## Introduction

Enteric fever (also known as typhoid or paratyphoid fever) is a systemic disease caused by *Salmonella enterica* serovars Typhi, Paratyphi A, Paratyphi B, and Paratyphi C (designated typhoidal *Salmonella* serovars), which are highly human-specific pathogens. These typhoidal *Salmonella* serovars have different epidemiological characteristics, clinical manifestations, and immune responses in human hosts, which are different from those of the non-typhoidal *Salmonella* among 2,579 *Salmonella* serovars [1-4]. Enteric fever, which can cause life-threatening illnesses with a high mortality rate, is responsible for numerous human infections worldwide and remains an important public health concern [2, 3, 5-9]. Research on enteric fever, particularly typhoid fever caused by *Salmonella* Typhi, mostly comprises studies on the epidemiology and clinical microbiology of infectious diseases. Additionally, interest in paratyphoid fever caused by *Salmonella* Paratyphi A, Paratyphi B, and Paratyphi C has been growing owing to the recent increased incidence rate worldwide and traveler outbreaks of paratyphoid fever in developed countries [2, 6, 8, 10].

General microbiological laboratory diagnostics for certain *Salmonella* serovars, including typhoidal *Salmonella* serovars, are culture-based serological methods that require a minimum of 4-5 days for reliable identification; additionally, these methods are labor intensive and expensive [11]. Early reliable diagnosis of enteric fever is critical for early surveillance, preventing the spread of salmonellosis, and timely medical treatment of patients, particularly for the infections of typhoidal *Salmonella* serovars [12, 13]. Currently, in clinical microbiology, representative, routine, and practical diagnostic methods for infectious diseases include culture-based methods combined with matrix-assisted laser desorption ionization-time of flight mass spectrometry (MALDI-TOF MS) analysis of blood, stool, or fluid specimens from patients [14]. Although MALDI-TOF MS analysis can be used to accurately identify the bacterial genus or species level, it is inefficient at the *Salmonella* serovar level and requires an additional culture-based serotyping test for conclusively diagnosing enteric fever, differentiated from other infectious pathogens or within *Salmonella* serovars. The low sensitivity of the recovery of *Salmonella* from clinical specimens and the resolution of MALDI-TOF MS analysis remain challenges for reliably diagnosing enteric fever clinically [5, 7, 8, 13, 15].

While DNA-based PCR detection methods for *Salmonella* Typhi [13, 16-19] and Paratyphi A [11, 20, 21] have been reported, few studies have reported similar methods for *Salmonella* Paratyphi B and Paratyphi C [22, 23]. Ultimately, these studies using DNA-based PCR detection methods must be applicable for enteric fever diagnostics in clinical microbiology. Despite the numerous advantages of PCR diagnostics, their efficiency of PCR diagnostics for *Salmonella* identification must be improved to provide more accurate results and prevent biased diagnostic conclusions at the *Salmonella* serovar level owing to the limited number of target genes and evaluated *Salmonella* serovars [5, 13, 20, 21, 23].

In our previous studies, PCR-based identification methods for *Salmonella* Typhimurium and Typhi were developed using specific genetic markers selected from comparisons among *Salmonella* genome sequences [24-26]. We inferred that PCR using appropriate genetic markers might sufficiently discriminate between specific *Salmonella* serovars. The present study aimed to develop reliable PCR diagnostics in a single reaction that could efficiently discriminate between typhoidal and non-typhoidal *Salmonella* serovars. Moreover, we aimed to employ the developed typhoidal *Salmonella* heptaplex PCR for efficiently diagnosing enteric fever in clinical microbiology laboratories. We believe that this method will enable rapid and reliable diagnosis of typhoidal *Salmonella* serovars and contribute to improving human health and public hygiene.

## Materials and Methods

### Bacterial Strains

A total of 200 *Salmonella* strains were used, including 112 serovars of *Salmonella* subspecies I-VI, as listed in Table 1. Sixteen type strains of *Salmonella* were obtained from the American Type Culture Collection (ATCC). Other *Salmonella* strains were obtained from the Federal Institute for Risk Assessment (BFR) of Germany [27], US Food and Drug Administration (FDA, CFSAN/OPDFB) [28], Korea Consumer Protection Board (KCPB) [29], Ministry of Food and Drug Safety (MFDS) of Korea, National Culture Collection for Pathogens (NCCP) of Korea, Food-borne pathogen Omics Research Center (FORC) of Korea, and Asian Bacterial Bank (ABB) of the Asia Pacific Foundation for Infectious Diseases (APFID) in Korea as listed in Table 1. *Salmonella* strains were inoculated in tryptic soy broth (TSB) and cultured at 37°C under vigorous shaking conditions.

### Genomic DNA Extraction

The cultured media of *Salmonella* strains were harvested in microtubes. Genomic DNA from *Salmonella* was extracted using a DNeasy Blood & Tissue kit (Qiagen, Germany) according to the manufacturer’s instructions. The concentration of the extracted DNA was measured using a UV spectrophotometer (model UV-1700; Shimadzu, Japan), and genomic DNA at a 1.8 to 2 ratio (*A_260_/A_280_*) was used. Genomic DNA from *Salmonella* strains was diluted in distilled water to 25 ng/μl and stored at 4°C prior to use in PCR.

### Genetic Markers for Typhoidal *Salmonella* Serovars and Primer Design

In our previous study, 195 genes of *Salmonella* Typhi CT18 (GenBank Accession No. NC_003198) were found to be highly specific to *Salmonella* genus and serovar Typhi [26]. These genes were subjected to the non-redundant (nr) DNA sequence database of the National Center for Biotechnology Information (NCBI, http://www.ncbi.nlm.nih.gov/) using the BLAST program [30] to screen for candidate genes specifically present in *Salmonella* Typhi and Paratyphi A, B, or C. Primers for screening candidate genes were designed and constructed (Bioneer, Korea).

### Single PCR Condition

PCR was performed using primers constructed from genomic DNAs of various *Salmonella* serovars, as listed in Table 1. Each 25 μl PCR mixture contained 1× EX Taq buffer, 0.4 μmol/l primer, 200 μmol/l concentrations of each dNTP, 0.5 Unit of EX Taq DNA polymerase (TaKaRa, Japan), and 25 ng/μl template DNA. PCR amplification was performed in a thermocycler (Model GeneAtlas G, ASTEC, Japan) with an initial denaturation at 94°C for 3 min, followed by 30 cycles of 94°C for 30 s, 65°C for 30 s, 72°C for 30 s and a final extension at 72°C for 3 min and 4°C for 5 min. Amplified products were electrophoresed on a 2% agarose gel in 0.5× Tris-Acetate–EDTA buffer, stained with DNA staining reagent (NEOscience, Korea), and photographed under UV-irradiation using a Vilber Gel Doc system (KoreaBIOMICS, Korea).

### Internal Amplification Control

To generate an Internal Amplification Control (IAC) for verifying of PCR performance, the partial DNA sequence of the tubulin β-4 chain gene (GenBank Accession No. NM_123801) was amplified from the genomic DNA of *Arabidopsis thaliana* using primers STM3098_F2_ flank_TB (5'-TTT GGC GCA GGC GAT TC-CAA TCC AGG AGA TGT TTA GGC G-3'), and STM3098_R2_ flank_TB (5'-GCC TCC GCC TCA ATC CG-CCT TTC TCC TGA ACA TAG CTG TG-3'), to generate a 100-bp amplicon. The resulting amplicon was inserted into pGEM-T Easy Vector (Promega Corporation, USA) to generate an IAC template plasmid for use in the typhoidal *Salmonella* heptaplex PCR.

### Typhoidal *Salmonella* Heptaplex PCR

Heptaplex PCR for typhoidal *Salmonella* serovars was performed using the primer sets listed in Table 2. Heptaplex PCR was designed to amplify eight genes targeting generic *Salmonella*, *S. enterica* subspecies *enterica* (I), *Salmonella* Typhi, Paratyphi A, Paratyphi B, Paratyphi C, and IAC. Heptaplex PCR was performed with seven primer sets at each concentration (Table 2) and IAC-template plasmid (approximately, 5 × 10^7^ copies) using AccuPower Multiplex PCR PreMix (K-2111, BIONEER, Korea) in a 20-μl mixture. The reaction conditions were the same as those mentioned in the previous section for single PCR, except initial denaturation at 94°C for 10 min. A amplified products were electrophoresed on a 3.5% agarose gel for 100 V, 70 min. The amplified products were analyzed using an Agilent 2100 Bioanalyzer (Agilent Technologies, USA) equipped with a DNA 1000 LabChip kit (Agilent Technologies).

### Preparation of *Salmonella*-Spiked Blood Culture Samples

Each culture of *Salmonella* Typhi and Paratyphi A, B, and C was serially diluted in TSB and then added to human whole blood at a concentration of 5 CFU/ml. For the blood culture, 10 ml of *Salmonella*-spiked blood was inoculated into Bact/ALERT SA Aerobic medium bottle (40 ml) (bioMérieux, INC., Durham, NC 27712, USA) and then cultured in a shaking incubator at 210 rpm at 37°C for up to 24 h. Human whole blood (SER-WB, Lot#: WB082819E) containing K2-EDTA as anticoagulant, which was obtained from a healthy volunteer adult donor who has signed an the Institutional Review Board (IRB) validated donor consent form, was purchased from the Zenbio Inc. (Research Triangle Park, NC 27709, USA). Cultured blood samples were collected at 0, 6, 9, 10.5, 12, 13.5, 15, 16.5, 18, 21, and 24-h time points. One milliliter of the collected blood culture was centrifuged at 16,000 ×*g* for 10 min, and the supernatant was carefully discarded. A total of 200 μl PrepMan Ultra reagent (Life Technologies, USA) was added, boiled for 15 min at 100°C, centrifuged for 5 min at 16,000 ×*g*, and finally 2 μl of supernatant was added as template DNA for the heptaplex PCR. Additionally, *Salmonella* colonies were recovered from the collected blood culture at the 6-h point. Blood culture (100 μl) was spread onto tryptic soy agar plate and cultured for 10 h at 37°C to allow *Salmonella* colony formation. A portion of *Salmonella* colony was picked using a sterile pipette tip and suspended in 100 μl PrepMan Ultra reagent or TE buffer (pH 8.0). Each sample was boiled for 15 min at 100°C, and centrifuged for 5 min at 16,000 ×*g*. Supernatant (2 μl) was added as template DNA for each typhoidal *Salmonella* heptaplex PCR. Also, a small portion of recovered *Salmonella* colony was picked using a sterile pipette tip and directly added into the heptaplex PCR (termed as “PCR on colony”).

## Results

### Selection of Genetic Marker for Constructing of Typhoidal *Salmonella* Heptaplex PCR

In our previous study on selecting novel genetic markers for *Salmonella* Typhi, 195 genes of *Salmonella* Typhi CT18 (GenBank Accession No. NC_003198) were screened via comparative genomics, which are present only in *Salmonella* genus [26]. We determined that some of the 195 genes were highly specific for *Salmonella* Typhi, Paratyphi A, Paratyphi B, or Paratyphi C. In the present study, candidate genetic markers were selected from 195 genes that were highly specific to *Salmonella* Paratyphi A, Paratyphi B, or Paratyphi C, based on the BLAST output against the NCBI nr database. Primer sets were designed for the selected 13 candidate genes and were evaluated with various genomic DNAs of *Salmonella* serovars to finalize the selection of specific genetic markers for identifying each *Salmonella* Paratyphi A, Paratyphi B, and Paratyphi C. Finally, a typhoidal *Salmonella* heptaplex PCR was constructed, including the selected genetic markers presented in Table 2. The heptaplex PCR included specific genetic markers for *Salmonella* genus (STM3098, 423 bp) [24, 26], *Salmonella* subspecies I (STM4057, 137 bp) [24, 26], *Salmonella* Typhi (STY1599, 258 bp) [26], Paratyphi A (STY3279, 193 bp and STY2750, 70 bp), Paratyphi B (STY3670, 165 bp), Paratyphi C (STY4578, 291 bp) and IAC (100 bp). IAC amplification was performed using primer set of STM3098.

### Specificity of Typhoidal *Salmonella* Heptaplex PCR

**Fig. 1:**
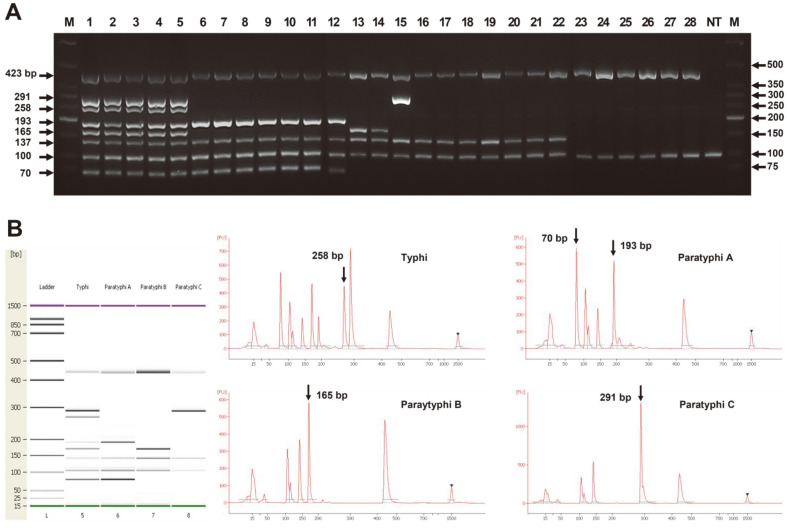
Performance of typhoidal *Salmonella* heptaplex PCR for detecting typhoidal *Salmonella* serovars. Panel A: Heptaplex PCR results with genomic DNAs from various *Salmonella* strains electrophoresed on a 3.5% agarose gel at 100 V for 70 min. M, DNA ladder; lane 1~5 *Salmonella* Typhi ATCC 33459, NCCP 14641, NCCP 10820, NCCP 12201, NCCP 10340; lane 6-12, *S*. Paratyphi A NCCP 14759, S11 (food isolate), 12-01 (clinical isolate), 12-02 (clinical isolate), 12-05 (clinical isolate), 12-07 (clinical isolate), 13-02 (clinical isolate); lane 13-14, *S*. Paratyphi B ATCC 10719, NCCP 12204; lane 15, *S*. Paratyphi C ATCC 13428; lane 16-18, S. Typhimurium ATCC 19585, ATCC 13311, ATCC 14028; lane 19, *S. choleraesuis* ATCC 13312; lane 20, *S*. Enteritidis ATCC 4931, lane 21, *S. gallinurum* ATCC 9184; lane 22, *S. pullorum* ATCC 9120; lane 23, *S*. subspecies salamae ATCC 15793; lane 24, *S*. subspecies arizonae ATCC 13314; lane 25, *S*. subspecies diarizonae ATCC 43973; lane 26, *S*. subspecies houtenae ATCC 43974; lane 27, *S. enterica* subspecies indica ATCC 43976; lane 28, *S. bongori* ATCC 43975, lane 29, no template DNA. Panel B: Analysis of heptaplex PCR results for *Salmonella* Typhi, Paratyphi A, Paratyphi B, and Paratyphi C using an Agilent Bioanalyzer 2100. The X-axis on the electropherogram represents the amplicon size (bp) and the Y-axis represents the fluorescence units (FUs). The arrow(s) showed the specific amplification marker(s) of each *Salmonella* serovar.

The developed typhoidal *Salmonella* heptaplex PCR assay was evaluated using 112 *Salmonella* serovars (200 strains), as shown in Table 1. The heptaplex PCR results demonstrated its specific diagnostics for *Salmonella* genus, *Salmonella* subspecies I, and *Salmonella* Typhi, Paratyphi A, Paratyphi B, and Paratyphi C, respectively as shown in Fig. 1 (panel A). Additionally, the PCR products were analyzed using capillary electrophoresis (Bioanalyzer), as shown in Fig. 1 (panel B). The specific peak(s) of each typhoidal *Salmonella* serovar [marked with arrows] demonstrated clean amplification of the expected size. All *Salmonella* Typhi strains showed amplification of all PCR products owing to the presence of all target genes in *Salmonella* Typhi. However, the 258 bp PCR product is a critical diagnostic indicator of *Salmonella* Typhi. *Salmonella* Paratyphi A strains, including clinical and food isolates, showed both amplifications of 193 and 70 bp, respectively. These two simultaneous amplifications are critical for diagnosing of *Salmonella* Paratyphi A, because some *Salmonella* serovars, such as serovars Georgia, Montevideo, Ohio, Muenster, and Kentucky, revealed one positive results between the two genetic markers. *Salmonella* Paratyphi B and Paratyphi C showed specific amplifications at expected sizes of 165 bp and 291 bp, respectively. As expected, all *Salmonella* strains showed *Salmonella* specific amplification at 423 bp and all strains belonging to *Salmonella* subspecies I showed specific amplification at 137 bp. In this study, amplification of IAC in all reactions removed of false negatives.

### Performance of Typhoidal *Salmonella* Heptaplex PCR with *Salmonella*-Spiked Blood Culture Sample

**Fig. 2:**
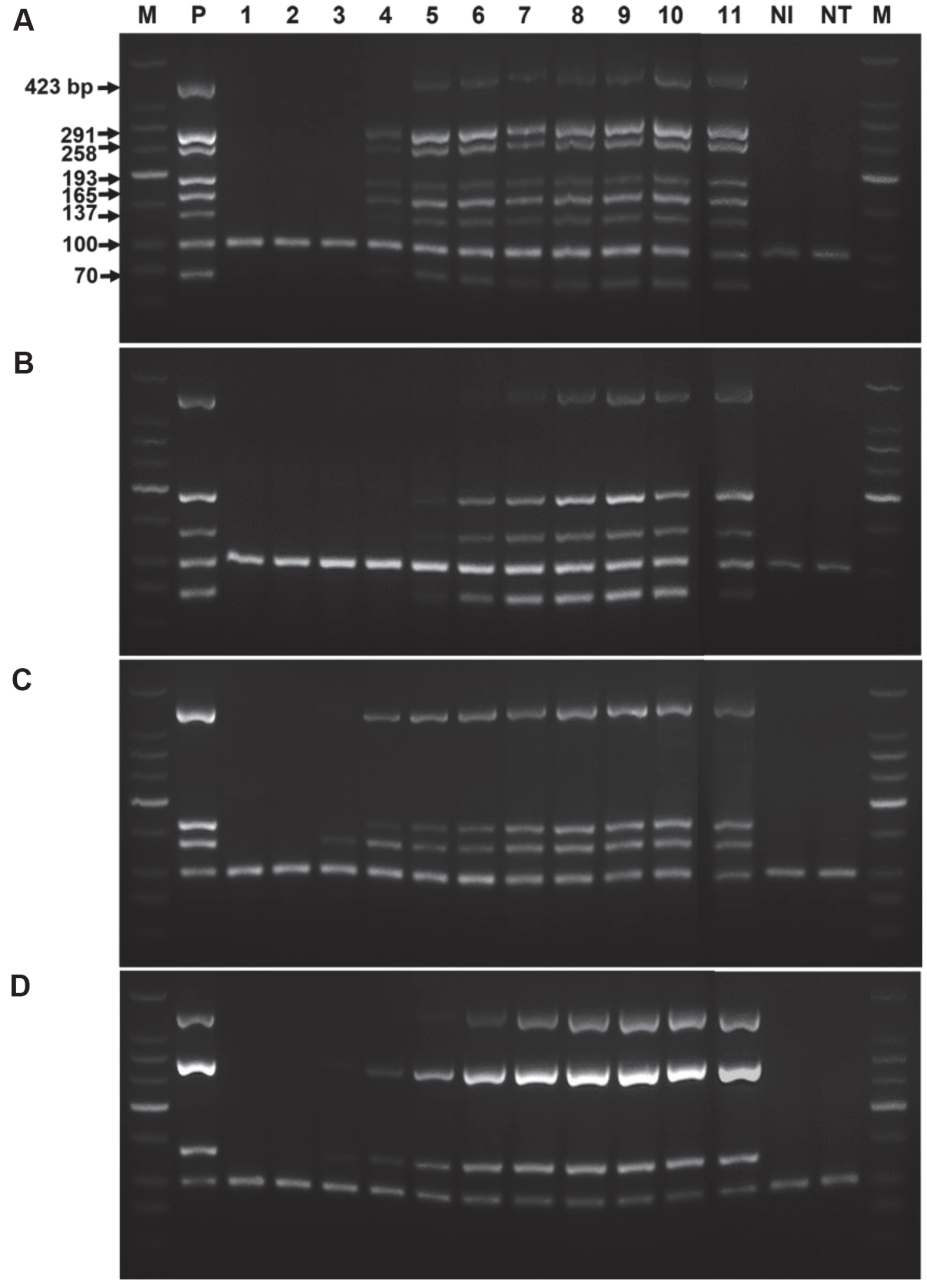
Performance of typhoidal *Salmonella* heptaplex PCR on *Salmonella*-spiked blood culture samples by culture time. Panel A: *Salmonella* Typhi, Panel B: *Salmonella* Paratyphi A, Panel C: *Salmonella* Paratyphi B, Panel D: *Salmonella* Paratyphi C. M: DNA ladder; P: positive control; lane 1~11: 0, 6, 9, 10.5, 12, 13.5, 15, 16.5, 18, 21, 24-h blood culture; NI: No Inoculation, 36-hour blood culture control without *Salmonella*; NT: No template.

**Fig. 3:**
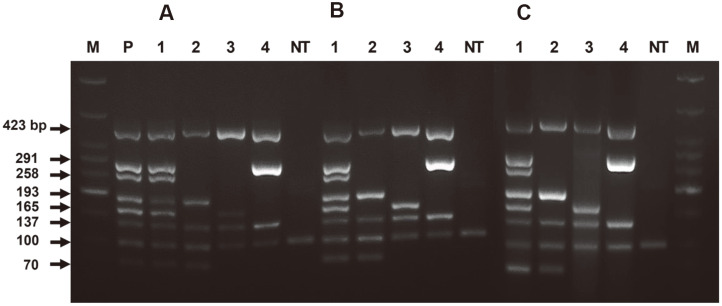
Diagnostic ability of typhoidal *Salmonella* heptaplex PCR on recovered *Salmonella* colonies from 6-h cultured blood samples inoculated with typhoidal *Salmonella* serovars. Panel A: DNA extraction using UltraPrepMan solution, Panel B: DNA extraction using TE buffer (pH 8.0), Panel C: PCR on colony. Lane 1: *Salmonella* Typhi; lane 2: *S*. Paratyphi A; lane 3: *S*. Paratyphi B, lane 4; *S*. Paratyphi C; lane M: DNA marker; P: positive control with genomic DNA of *Salmonella* Typhi; NT: No template DNA.

The developed typhoidal *Salmonella* heptaplex PCR was employed for existing blood culture systems, which are used globally for diagnosing various infectious diseases, including enteric fever, in clinical microbiology. The extracted DNA solutions from *Salmonella*-spiked blood culture samples were evaluated as shown in Fig. 2. Blood culture samples spiked with *Salmonella* Typhi, Paratyphi A, Paratyphi B, and Paratyphi C showed positive heptaplex PCR results after 12- or 13.5-h of blood culture. Additionally, recovered colonies from *Salmonella*-spiked blood cultures at the 6-h point (PCR on colony) and extracted DNA from recovered colonies using two boiling methods were evaluated using typhoidal *Salmonella* heptaplex PCR, as shown in Fig. 3. The two boiling methods provided clean amplification with each typhoidal serovar (Fig. 3 panels A and B), and direct PCR on the colony of each typhoidal serovar recovered from blood culture also showed clean amplification (Fig. 3 panel C). These results demonstrate that the developed typhoidal *Salmonella* heptaplex PCR could be applied to existing blood culture systems in clinics to obtain detailed serovar information among typhoidal *Salmonella* serovars.

## Discussion

Generally, *Salmonella* spp., particularly *Salmonella* subspecies I, are considered pathogens of birds and mammals, including humans, despite some host-specific *Salmonella* serovars. Therefore, identification of *Salmonella* at the genus and subspecies levels is important for diagnosing salmonellosis in clinics and public hygiene. The typhoidal *Salmonella* heptaplex PCR developed in this study included two previously described genetic markers (STM3098 and STM4057 gene) for identifying the genus *Salmonella* and *Salmonella* subspecies I, respectively [24, 26]. These genetic markers could provide critical diagnostic information at *Salmonella* genus and subspecies levels against other infectious pathogens such as pathogenic *E. coli*. For diagnosing typhoidal *Salmonella* serovars, heptaplex PCR contains newly developed genetic markers for *Salmonella* Paratyphi A (STY3279 and STY2750), Paratyphi B (STY3670), and Paratyphi C (STY4578) and a marker for S. Typhi (STY1599) [26]. These genetic markers provide specific diagnostic tools for each typhoidal *Salmonella* serovars.

PCR-based diagnostics can be used to directly identify *Salmonella* Typhi in blood samples [16, 18, 31-33]. However, in these studies, direct DNA extraction from blood without culture (briefly termed “PCR on blood”) did not provide stable PCR amplification and sufficient analysis resolution to confirm positive results on agarose gel electrophoresis, which is not suitable for practical application in clinical microbiology. These difficulties may be because of the low number of *Salmonella* Typhi present in blood samples (0.5-22 CFU/ml) [34] and the failure to recover the genomic DNA of *Salmonella* directly from the blood sample. To overcome difficulties in early clinical diagnosis enteric fever from human blood samples, a PCR-based method can be used in the existing diagnostic systems in clinical microbiology, particularly in blood culture-based MALDI-TOF MS system [14].

Interestingly, the addition of ox-bile to the blood culture medium (generally TSB medium) enhances the growth rate of *Salmonella*, inhibiting the bactericidal activity of blood [35]. A single amplification PCR method for *Salmonella* Typhi was applied to a blood culture sample in ox bile-containing TSB medium, demonstrating clean positive results for a 5-h blood culture sample [34]. However, in the present study, the ox-bile-containing blood culture method was not employed because the presence of ox-bile in the blood culture medium only allowed the growth of bile resistant bacteria [13]. This method is not preferable in existing blood culture-based MALDI-TOF MS diagnostics, because it is a universal diagnostic tool for enteric fever and other infectious diseases in clinical microbiology. However, our heptaplex PCR revealed positive results for 12- or 13.5-h cultured blood samples, as shown in Fig. 2, which could provide early diagnostics for typhoidal *Salmonella* serovars. We agree that successful PCR-based diagnostics of typhoidal *Salmonella* in blood depend on the growth rate and number of *Salmonella* in blood cultures [34]. Additionally, PCR results of the recovered colony from the 6-h blood culture (Fig. 3) could provide complementary diagnostics at *Salmonella* serovar level along with the MALDI-TOF MS system in clinics.

Therefore, a simple diagnostic tool for enteric fever must be developed [5, 8, 13]. In the present study, a typhoidal *Salmonella* heptaplex PCR with novel genetic markers was developed and evaluated using various *Salmonella* serovars, demonstrating its performance and specificity for typhoidal *Salmonella* serovars. Moreover, the performance of this heptaplex PCR was validated using recovered colonies as well as directly extracted DNA from blood culture samples. The results demonstrated that this typhoidal *Salmonella* heptaplex PCR provides a novel, reliable DNA-based diagnostic tool for *Salmonella* typhoidal serovars related to public hygiene, including in the fields of clinical microbiology, food safety, and epidemiology, and could potentially help in early diagnosis of enteric fever when combined with existing blood culture processes in clinics.

## Figures and Tables

**Table 1:** Salmonella strains used in this study and their results with typhoidal *Salmonella* heptaplex PCR.

Salmonella subspecies and serovars (No.^a^)	Strain Designation or source^b^	Heptaplex PCR result^c^						
		STM3098	STM4057	STY1599	STY3279	STY2750	STY3670	STY4578
		*Salmonella* genus	*Salmonella* ssp. I	Typhi	Paratyphi A	Paratyphi A	Paratyphi B	Paratyphi C
*S. enterica* subspecies *enterica* (I)								
Aberdeen	NCCP 10142	+	+	-	-	-	-	-
Agona (4)	BFR, MFDS 1004876, KCPB	+	+	-	-	-	-	-
Agona B	FDA	+	+	-	-	-	-	-
Anatum	FDA	+	+	-	-	-	-	-
Bardo	NCCP 13572	+	+	-	-	-	-	-
Bareilly (2)	FDA, MFDS 1010896	+	+	-	- or +	-	-	-
Blockley	BFR	+	+	-	-	-	-	-
Bovismorbificans (2)	BFR, NCCP 12244	+	+	-	-	-	-	-
Braenderup (2)	MFDS 1008393, FDA	+	+	-	-	-	-	-
Brandenburg	BFR	+	+	-	-	-	-	-
Bredeney (2)	BFR, FDA	+	+	-	-	-	-	-
Brezany	NCCP 11678	+	+	-	-	+	-	-
California	FDA	+	+	-	-	-	-	-
Cerro	FDA	+	+	-	-	-	-	-
Choleraesuis	ATCC 13312	+	+	-	-	-	-	-
Derby (3)	BFR, MFDS 1009813, FDA	+	+	-	-	-	-	-
Dublin	BFR	+	+	-	-	-	-	-
Edinburg	KCPB	+	+	-	-	+	-	-
Elisabethville	NCCP 14030	+	+	-	-	-	-	-
Enteritidis (30)	ATCC 4931, FORC_019, FORC_052, FORC_051, KCPB, FDA	+	+	-	-	-	-	-
Essen	NCCP 13569	+	+	-	-	-	-	-
Gallinarum	ATCC 9184	+	+	-	-	-	-	-
Georgia (2)	KCPB	+	+	-	-	+	-	-
Give	NCCP 13696	+	+	-	-	+	-	-
Give E1	FDA	+	+	-	-	-	-	-
Goettingen	NCCP 11681	+	+	-	-	-	-	-
Haardt (5)	KCPB	+	+	-	-	-	-	-
Hadar (2)	BFR, KCPB	+	+	-	-	-	-	-
Havana	NCCP 12216	+	+	-	-	-	-	-
Heidelberg (3)	BFR, FDA	+	+	-	-	-	-	-
Hillingdon	NCCP 13574	+	+	-	-	-	-	-
Illinois	FDA	+	+	-	-	-	-	-
Indiana	NCCP 11669	+	+	-	-	-	-	-
Infantis (4)	BFR, MFDS 1010567, KCPB, FDA	+	+	-	-	-	-	-
Isangi	NCCP 14031	+	+	-	+	-	-	-
Istanbul (2)	NCCP 11684, KCPB	+	+	-	-	-	-	-
Java B	FDA	+	+	-	-	-	-	-
Javiana	FDA	+	+	-	-	+	-	-
Joal	KCPB	+	+	-	-	+	-	-
Kedougou	NCCP 11685	+	+	-	-	-	-	-
Kentucky	FDA	+	+	-	-	+	-	-
Kottbus	NCCP 12234	+	+	-	-	-	-	-
Lindenburg	NCCP 11687	+	+	-	-	-	-	-
Litchfield (2)	BFR, FDA	+	+	-	-	-	-	-
Livingstone (2)	BFR, MFDS 1004819	+	+	-	-	-	-	-
London	MFDS 1004861	+	+	-	-	-	-	-
Madelia	FDA	+	+	-	-	-	-	-
Manhattan	FDA	+	+	-	-	+	-	-
Mbandaka (2)	FORC_015, FDA	+	+	-	-	-	-	-
Meleagridis	FDA	+	+	-	+	-	-	-
Mhenohen	FDA	+	+	-	-	-	-	-
Mississippi	FDA	+	+	-	-	-	-	-
Montevideo (5)	NCCP 10140, NCCP 12211, FDA, BFR, MFDS 1006814,	+	+	-	-	+	-	-
Muenster	FDA	+	+	-	-	+	-	-
Nchanga	NCCP 11693	+	+	-	-	-	-	-
Newport (2)	BFR, FORC_020	+	+	-	-	-	-	-
Nigeria	MFDS 1004862	+	+	-	-	-	-	-
Ohio (2)	MFDS 1008118, FDA	+	+	-	+	-	-	-
Oranienburg	FDA	+	+	-	-	-	-	-
Othmarschen	NCCP 13706	+	+	-	+	-	-	-
Panama	MFDS 1004857	+	+	-	-	+	-	-
Paratyphi A (7)	KCPB, ABB, NCCP 14759	+	+	-	+	+	-	-
Paratyphi B (2)	ATCC 10719, NCCP 12204	+	+	-	-	-	+	-
Paratyphi C	ATCC 13428	+	+	-	-	-	-	+
Planckendael	NCCP 11699	+	+	-	-	-	-	-
Poona	FDA	+	+	-	-	-	-	-
Pullorum	ATCC 9120	+	+	-	-	-	-	-
Reading	MFDS 1007899	+	+	-	-	-	-	-
Rissen	MFDS 1004867	+	+	-	+	-	-	-
Saintpaul (2)	FORC_058, BFR	+	+	-	-	-	-	-
Sandow	KCPB	+	+	-	-	-	-	-
Schwarzengrund (2)	MFDS 1006893, KCPB	+	+	-	-	-	-	-
Senftenberg	BFR	+	+	-	-	-	-	-
Singapore	NCCP 12218	+	+	-	-	+	-	-
Stanley	MFDS 1004865	+	+	-	-	+	-	-
Tennessee	KCPB	+	+	-	-	-	-	-
Thompson	MFDS 1006817	+	+	-	-	-	-	-
Tibati	NCCP 11703	+	+	-	-	-	-	-
Travis	NCCP 11705	+	+	-	-	-	-	-
Tumodi	NCCP 11706	+	+	-	-	-	-	-
Typhi (5)	ATCC 33459, NCCP 14641, NCCP 10820, NCCP 12201, NCCP 10340	+	+	+	+	+	+	+
Typhimurium (10)	ATCC 19585, ATCC 14028, ATCC 13311, BFR, KCPB, FORC_030	+	+	-	-	-	-	-
Vinohrady	NCCP 12217	+	+	-	-	-	-	-
Virchow (3)	MFDS 1004870, BFR, FORC_038	+	+	-	-	-	-	-
Virginia (5)	KCPB	+	+	-	-	-	-	-
Weltevreden	NCCP 12239	+	+	-	-	+	-	-
4,[5],12:i:-	MFDS 1004858	+	+	-	-	-	-	-
*S. enterica* subspecies *salamae* (II)								
30:l,z28:z6	BFR	+	-	-	-	-	-	-
42:b:e,n,x,z15	BFR	+	-	-	-	-	-	-
42:r:-	BFR	+	-	-	-	-	-	-
48:d:z6	BFR	+	-	-	-	-	-	-
9,12:z:z39	BFR	+	-	-	-	-	-	-
9,46:z4,z24:z39:z 42	ATCC 15793	+	-	-	-	-	-	-
*S. enterica* subspecies *arizonae* (IIIa)								
18:z4,z32:-	BFR	+	-	-	-	-	-	-
21:g,z51:-	BFR	+	-	-	-	-	-	-
47:r:-	BFR	+	-	-	-	-	-	-
51:z4,z23:-	ATCC 13314	+	-	-	-	-	-	-
*S. enterica* subspecies *diarizonae* (IIIb)								
6,7:l,v:z53	ATCC 43973	+	-	-	-	-	-	-
18:i,v:z	BFR	+	-	-	-	-	-	-
47:l,v:z	BFR	+	-	-	-	-	-	-
50:z:z52	BFR	+	-	-	-	-	-	-
*S. enterica* subspecies *houtenae* (IV)								
11:z4,z23:-	BFR	+	-	-	-	-	-	-
16:z4,z32:-	BFR	+	-	-	-	-	-	-
45:g,z51:-	ATCC 43974	+	-	-	-	-	-	-
48:g,z51:-	BFR	+	-	-	-	-	-	-
*S. enterica* subspecies *bongori* (V)								
66:z41:-	ATCC 43975	+	-	-	-	-	-	-
44:r:-	BFR	+	-	-	-	-	-	-
48:z35:-	BFR	+	-	-	-	-	-	-
66:z65:-	BFR	+	-	-	-	-	-	-
*S. enterica* subspecies *indica* (VI)								
1,6,14,25:a:e,n,x (2)	ATCC 43976, BFR	+	-	-	-	-	-	-
41:b:1,7	BFR	+	-	-	-	-	-	-
45:a:e,n,x	BFR	+	-	-	-	-	-	-
Total (112 serovars)	200 strains							

^a^No., Number of strains.

^b^BFR, Federal Institute for Risk Assessment; KCPB, Korea Consumer Protection Board; FDA, US Food and Drug Administration (CFSAN/OPDFB); MFDS (Ministry of Food and Drug Safety); NCCP (National Culture Collection for Pathogens); FORC (Food-borne pathogen Omics Research Center); ABB (Asian Bacterial Bank) of APFID (Asia Pacific Foundation for Infectious Diseases).

^c^+, Positive result; -, negative result.

**Table 2:** Primer pairs used for typhoidal *Salmonella* heptaplex PCR and their expected result with typhoidal *Salmonella* serovars.

Target gene (synonym)	Primer	Primer sequence (5' --- 3')	Primer concentration (μmol/l)	PCR product size (bp)	Target *Salmonella* serovars or subspecies	Expected results with typhoidal *Salmonella* serovars^a^				References
						Typhi	Paratyphi A	Paratyphi B	Paratyphi C	
STM3098	STM3098 -F3	TTTRG CGGCR CAGGC GATTC	1	423	Genus *Salmonella*	+	+	+	+	[24,26]
	STM3098 -R3	GCCTC CGCCT CATCA ATYCG								
STY4578	STY4578 F32	CATTTCTGAGATTTAT TCTGACGCTTGTG	0.5	291	Typhi, Paratyphi C	+	-	-	+	In this study
	STY4578 R322	CTGAATATTCGCAAA TCGCGACG								
STY1599	STY1599 F	TTACC CCACA GGAAG CACGC	0.15	258	Typhi	+	-	-	-	[26]
	STY1599 R2	CTCGT TCTCT GCCGT GTGGG								
STY3279	STY 3279 F102	AATCA GCAGT GCGTT GAGAA AACC	1	193	Typhi, Paratyphi A	+	+	-	-	In this study
	STY3279 R294	GGAGT TAATA AGTGA TAGGA ACATT GTACT TACTG T								
STY3670	STY3670 47F	CCTTGGCTGGATGTG CTTTG	0.75	165	Typhi, Paratyphi B	+	-	+	-	In this study
	STY3670 211R	AGCCAGGAACTTCGT CACTC								
STM4057	STM4057 F3	GGTGG CCTCS ATGAT TCCCG	0.375	137	*Salmonella* subspecies *enterica* (I)	+	+	+	+	[24,26]
	STM4057 R	CCCAC TTGTA GCGAG CGCCG								
STY2750	STY2750 F7	TTTCT GTGTA GYGCA CAGCT TCTGG C	0.75	70	Typhi, Paratyphi A	+	+	-	-	In this study
	STY2750 R76	TGCTG CCAGT GAAAC CCACT ATTGT GTCG								
IAC^b^	-	-	100			+	+	+	+	

^a^+, Positive result; -, Negative result

^b^IAC, Internal amplification control in heptaplex PCR
